# Current Clinical Practices for Gaming Disorder and Related Issues in Primary Care Settings in Northern Japan: A Regional Survey in Sapporo

**DOI:** 10.7759/cureus.80721

**Published:** 2025-03-17

**Authors:** Masaru Tateno, Yukie Tateno, Tomohiro Shirasaka, Hirofumi Furuta, Yoshito Takahashi

**Affiliations:** 1 Child Mental Health Clinic of Neuropsychiatry, Sapporo Medical University Hospital, Sapporo, JPN; 2 Tokiwa Child Development Center, Tokiwa Hospital, Sapporo, JPN; 3 Psychiatry, Teine Keijinkai Medical Center, Sapporo, JPN; 4 Pediatrics, Furuta Pediatric Clinic, Sapporo, JPN; 5 Psychiatry, Soen Mental Clinic, Sapporo, JPN

**Keywords:** attention deficit hyperactivity disorder (adhd), autism spectrum disorder, behavioral addiction, gaming disorder, internet addiction, internet gaming disorder, neurodevelopmental disorder, psychiatric comorbidity

## Abstract

Background: Gaming is a popular leisure activity among children and adolescents in Japan. Recently, gaming disorder (GD) has become a common issue in child and adolescent psychiatry. However, due to a severe shortage of child and adolescent psychiatrists (CAPs) in Japan, we hypothesized that young patients with GD and gaming-related problems may seek help at pediatric or general psychiatric clinics, which serve as primary care providers for children's mental health issues. In this study, we conducted a regional survey in Sapporo, Japan, to understand the current status of medical interventions for GD provided by pediatricians and psychiatrists.

Methods: This was a cross-sectional survey conducted in a specific region of Japan. The subjects of this study were almost all pediatricians, psychiatrists, and CAPs practicing in Sapporo, Japan. A questionnaire was mailed to a total of 196 medical institutions. The survey included questions about clinical practices related to GD, such as experience in treating GD, common presenting symptoms in patients with GD, and psychiatric comorbidities associated with GD. Results were statistically compared between two groups: pediatricians and psychiatrists.

Results: We received 60 responses, yielding a response rate of 30.6%. All collected response forms were considered valid, and only one response to the question about the percentage of patients with gaming-related problems was considered invalid because it was an outlier (answer of 80%). The rate of respondents who had encountered gaming-related problems was 53.8% (n=14) among pediatricians (n=26), 52.2% (n=12) among psychiatrists (n=23), and, as a reference, 81.8% (n=9) among CAPs (n=11). When asked about the proportion of patients with gaming-related problems among all the patients they had seen, pediatricians reported 0.6%, psychiatrists reported 2.9%, and CAPs reported 8.7%. Autism spectrum disorder (ASD) was the most commonly reported comorbid diagnosis associated with GD, followed closely by attention deficit hyperactivity disorder (ADHD). Depression and anxiety were the third most commonly reported comorbid conditions.

Discussion: While many CAPs (81.8%) are already addressing gaming-related issues, only about half of pediatricians (53.8%) and psychiatrists (52.2%) are involved in such treatment. With the increasing number of children and adolescents engaging in online gaming, playtime has gradually increased, potentially leading to an increase in GD cases. The difference was that pediatricians were often consulted about somatic symptoms, while psychiatrists were typically required to manage behavioral issues, such as excessive spending on gaming, and psychiatric symptoms, such as anxiety. As the number of children and adolescents seeking medical care for gaming-related problems is expected to increase, it is anticipated that clinical guidelines will be developed to enable both pediatricians and psychiatrists to effectively manage these cases.

## Introduction

Gaming is one of the most popular leisure activities among youth in Japan, especially among adolescent males [1,2]. The number of Internet users in Japan continues to grow, while the age at which children start using the Internet continues to decline. According to the Survey on the Internet Use Environment of the Youth in Japan, based on parents' responses, the percentage of children aged one who use the Internet daily increased from 33.1% in the 2023 survey (n=2,160) to 41.9% in the 2024 survey (n=1,964) [3]. Similarly, for children aged three, the percentage rose from 58.7% to 72.6%. As the number of children using the Internet increases, the number of Internet-related problems has also increased.

A large-scale survey in Japan reported that children start using the Internet in early childhood by watching videos on their parents' smartphones and tablets [3]. As they grow older and enter elementary school, they begin to connect game consoles to the Internet and enjoy playing online games. Today, most school-age children play online games with their peers [4]. Parents recognize that prolonged gaming can negatively impact their children's mental and physical health. As a result, concerns about excessive gaming are increasingly being brought up during medical visits for other reasons. However, the actual state of medical care for this issue remains unclear.

The inclusion of gaming disorder (GD) as a psychiatric disorder in the 11th edition of the International Classification of Diseases (ICD-11) is attracting public attention through the mass media [5]. Among several forms of addiction, gaming disorder is unique in that most patients are children and adolescents. Many parents are concerned about their children's excessive gaming and related mental health issues [6]. Sensationalized media coverage and excessive societal reactions may be causing unnecessary anxiety among parents whose children engage in gaming in a healthy manner. This, in turn, may be driving some parents to seek medical consultations. However, the current state of clinical practice remains unclear.

We previously conducted a survey of child and adolescent psychiatrists (CAPs) in Japan to understand the clinical practice for GD and to investigate what kind of difficulties they were experiencing [7]. Our survey results demonstrated that many CAPs who deal with adolescents' mental health issues are often consulted for gaming-related problems. However, none of the respondents provided specialized treatment to GD at that time. In Japan, it has been reported that many child and adolescent patients visiting medical institutions regularly had neurodevelopmental disorders (NDD) such as autism spectrum disorder (ASD) and attention deficit hyperactivity disorder (ADHD) [8]. There is a chronic and severe shortage of CAPs in Japan, leading to an exceedingly long waiting list for consultations [9].

The healthcare system in Japan ensures that all residents are free to visit any medical institution of their choice without a referral from a primary care physician. Thus, we hypothesized that patients with GD, which seems to be more common among young people, may visit pediatricians and general psychiatrists. Although public interest in GD is high, the current state of clinical practice remains unclear. In a previous national survey of CAPs, we found that many of them were already treating GD [8]. The aim of this study is to investigate the current clinical practice of GD treatment in both pediatrics and psychiatry.

The ICD-11 officially came into effect in January 2022, defining GD as a psychiatric disorder. However, there is still limited information on how GD is being treated in clinical practice. Given the extremely long waiting lists for child and adolescent psychiatry consultations in Japan, we conducted a survey of pediatricians and general psychiatrists who serve as primary care physicians for children's mental health. Understanding whether there is already a need to treat children and adolescents with GD-related issues outside of child and adolescent psychiatry services would be valuable. The aim of this study is to collect information on the clinical practice of GD in Sapporo, Japan, in preparation for a future national survey on the clinical management of GD in pediatrics and general psychiatry. In this study, we conducted a regional survey on the current status of medical intervention for GD in Sapporo as a preliminary survey before the national survey planned in the near future.

## Materials and methods

The subjects of this study were almost all pediatricians, general psychiatrists, and CAPs practicing in Sapporo, Japan. Sapporo has the fourth largest population of any city in Japan (approximately 1.97 million) and forms a metropolitan area with almost 40% of the total population of Hokkaido prefecture (approximately 5.11 million).

The questionnaire was mailed to the director or department head of each clinic or hospital (Appendix 1 and 2). To enhance the response rate, we sent follow-up messages via the mailing lists of pediatricians and psychiatrists before the submission deadline.

This study was a regional survey that essentially included all pediatric and psychiatric institutions. However, since only a few medical institutions may not be affiliated with an association, this study specified that it did not cover all pediatric and psychiatric institutions, but only those affiliated with the following associations: members of the Sapporo Pediatric Association (including both clinics and hospitals) (n=104), the Sapporo Branch of the Association of Psychiatric Clinics in Hokkaido (n=57), member hospitals of the Hokkaido Branch of the Japan Psychiatric Hospitals Association located in Sapporo (n=30), and General/University hospitals that are not members of those association (n=5).

The subjects were requested to complete the questionnaire anonymously and return it in the provided envelope. The envelope contained a two-page questionnaire, a cover letter explaining the purpose of the study, details about the consent process, and a request to hand the questionnaire to a doctor specializing in GD if they knew one at their hospital.

The questionnaire collected information on the respondents' socio-demographic characteristics and the estimated prevalence of patients with gaming-related problems, including those potentially meeting the criteria for GD. It also included questions about the most common and primary complaints of patients suspected of excessive gaming at their initial visit, the therapeutic interventions used at each institution, and common psychiatric comorbidities associated with GD.

Since it was assumed that many subjects would not be familiar with the diagnostic guidelines for GD in ICD-11 [5], the following explanation was provided at the beginning of the questionnaire: “In the ICD-11, gaming disorder is defined as a dysfunctional pattern of gaming, characterized by: a) impaired control; b) an increasing priority given to gaming; and c) a continued involvement in gaming despite negative consequences. The maladaptive gaming pattern has to be manifested over an extended period of time (typically 12 months), and cause significant impairment in social functioning.”

The responses were collected between December 1, 2024 and January 17, 2025.

A chi-square test was conducted to compare the frequencies of clinical symptoms and psychiatric comorbidities of patients with GD between two groups: pediatricians and psychiatrists. Statistical analyses were performed using StatFlex Ver.7 (Artec Inc., Osaka, Japan). Statistical significance was set at P < 0.05.

The study's aim was clearly stated on the cover page and answering the questionnaire was deemed to constitute consent. The study protocol was approved by the ethics committee of Tokiwa Hospital (TH-241023). The study procedures were carried out in accordance with the Declaration of Helsinki.

## Results

We mailed the questionnaire to a total of 196 medical institutions and received 60 responses, resulting in a response rate of 30.6%. Table 1 summarizes the results of the questions regarding the respondents' socio-demographic characteristics and their experience in treating GD. 

**Table 1:** Socio-demographic Characteristics of Respondents and Their Experience in the Treatment of Gaming Disorder

		Pediatricians (n=26)	General Psychiatrists (n=23)	Child and Adolescent Psychiatrists (n=11)
Place of work	Clinic	15	12	8
	Hospital	11	11	3
Age group(s) of patients seen (checkbox)	Infant/Pre-school	26	0	7
	Elementary school student	26	0	9
	Junior high school student	26	5	11
	High school students and equivalent	14	10	11
	Adults	4	23	7
Percentage of respondents who have provided treatment for gaming disorder (n)		53.8 (14)	52.2 (12)	81.8 (9)
Estimated percentage of patients presenting with gaming-related problems (%)		0.6	2.9	8.7
Estimated prevalence of patients with gaming disorder (%)		0.1	1.6	1.6
Currently feasible treatments for gaming disorder (multiple answers allowed)				
Individual therapy by a physician		6	7	3
Counseling by a clinical psychologist		1	4	2
Group therapy		0	2	0
Family support (family meetings, group therapy etc.)		0	0	0
Day care (day hospital)		0	1	0
No treatment programs for gaming disorder		15	15	8
Blank		5	1	0
Others				
Conditioned reflex control (individual therapy)		1	0	0
Inpatient care/hospitalization		2	0	1
Referral to other institutions		1	0	0

Among pediatricians (n=26), 53.8% (n=14) reported seeing high school students. In contrast, only 21.7% (n=5) of psychiatrists (n=23) saw junior high school students, while 43.5% (n=10) saw high school students. Notably, all CAPs indicated that they treated both junior high and high school students. The proportion of respondents who had dealt with gaming-related problems was 53.8% (n=14) among pediatricians (n=26), 52.2% (n=12) among psychiatrists (n=23), and 81.8% (n=9) among CAPs (n=11).

When asked about the percentage of patients with gaming-related problems among all the patients they had seen, pediatricians reported 0.6%, psychiatrists reported 2.9%, and CAPs reported 8.7%. One CAP reported that 80% of their patients had gaming-related problems; however, this figure was significantly higher than the other responses and was considered a likely typographical error. Furthermore, the interquartile range (IQR) was calculated, and 80 was identified as an outlier. As a result, it was excluded from statistical analyses. Additionally, when asked about the percentage of patients diagnosed with GD, pediatricians reported 0.1%, while both psychiatrists and CAPs reported 1.6%.

When asked about treatment for GD, 76.9% (n=20) of pediatricians (n=26), 69.6% (n=16) of psychiatrists (n=23), and 72.7% (n=8) of CAPs (n=11) reported that they did not provide any specific treatment for the condition.

Table 2 summarizes the responses regarding the clinical symptoms of patients with GD at the time of consultation. The most commonly reported symptoms were disturbances in the sleep-wake rhythm and school refusal/absenteeism. A chi-square test was conducted to compare the primary complaints observed in pediatric and psychiatric settings. A statistically significant difference was found only in overspending on online game transactions. Although no significant differences were observed, pediatricians tended to see more patients with somatic complaints, such as headaches, dizziness, and abdominal pain, compared to psychiatrists.

**Table 2:** Clinical Symptoms of Patients with Gaming Disorder at the Time of Initial Consultation An asterisk (*) indicates statistical significance at P < 0.05.

	Pediatricians (n=26)	General Psychiatrists (n=23)	Child and Adolescent Psychiatrists (n=11)	A chi-square test between Pediatricians and General Psychiatrists
Disturbed sleep-wake rhythm	15	11	9	χ²(1, N = 49) = 0.4770, P = 0.4898, φ = 0.0987
School refusal/absenteeism	14	10	9	χ²(1, N = 49) = 0.5250, P = 0.4687, φ = 0.1035
Academic decline	7	8	6	χ²(1, N = 49) = 0.3549, P = 0.5514, φ = -0.0851
Depression and anxiety	5	7	8	χ²(1, N = 49) = 0.8284, P = 0.3627, φ = -0.1300
Yelling and violence	4	5	9	χ²(1, N = 49) = 0.3287, P = 0.5664, φ = -0.0819
Overspending on gaming	0	9*	8	χ²(1, N = 49) = 12.4630, *P = 0.0004, φ = -0.5043
Somatic symptoms	7	3	3	χ²(1, N = 49) = 1.4474, P = 0.22895, φ = 0.1719

Table 3 presents the results of the survey on psychiatric disorders that frequently co-occur with GD. ASD was the most commonly reported comorbid diagnosis, followed closely by ADHD. Depression and anxiety were the third most frequently reported co-morbid conditions, with an equal number of responses. When comparing the results between pediatricians and psychiatrists, psychiatrists were significantly more likely to encounter patients with anxiety as a comorbidity of GD.

**Table 3:** Psychiatric Comorbidities Associated with Gaming Disorder ADHD: Attention Deficit Hyperactivity Disorder OCD: Obsessive-compulsive disorder An asterisk (*) indicates statistical significance at P < 0.05.

	Pediatricians (n=26)	General Psychiatrists (n=23)	Child and Adolescent Psychiatrists (n=11)	A chi-square test between Pediatricians and General Psychiatrists
Autism Spectrum Disorder	7	8	9	χ²(1, N = 49) = 0.3549, P = 0.5514, φ = -0.0851
ADHD	7	7	8	χ²(1, N = 49) = 0.0737, P = 0.7860, φ = -0.0388
Depression	3	5	3	χ²(1, N = 49) = 0.9296, P = 0.3350, φ = -0.1377
Anxiety	1	7*	3	χ²(1, N = 49) = 6.3156, *P = 0.0120, φ = -0.3590
Intellectual Disability	3	1	2	χ²(1, N = 49) = 0.8417, P = 0.3589, φ = 0.1311
Gambling Disorder	0	2	0	χ²(1, N = 49) = 2.3571, P = 0.1247, φ = -0.2193
OCD	0	1	0	χ²(1, N = 49) = 1.1540, P = 0.2827, φ =-0.1535

## Discussion

In our nationwide survey of CAPs in Japan, 83.0% (132 out of 159) reported experience of treating GD [8]. In this survey, 81.8% of CAPs (nine out of 11) had experience of treating this condition. These findings suggest that GD has become a common issue in the clinical practice of child and adolescent psychiatry.

When asked about the percentage of patients with gaming-related problems among all those they treated, pediatricians reported 0.6%, psychiatrists reported 2.9%, and CAPs reported 8.7%. Consistent with previous studies, it has been demonstrated that CAPs were found to have more frequent opportunities to treat patients with gaming-related issues.

There is a chronic shortage of CAPs in Japan, leading to long waiting times for initial consultations [9]. Given this situation, we hypothesized that pediatricians, who serve as primary care physicians for children's mental health issues, might play a role in treating GD. However, only 53.8% (14 out of 26) of pediatricians had experience of treating GD, and the proportion of pediatric patients diagnosed with GD was lower than we expected, accounting for just 0.1% of all pediatric patients seen by the respondents. Furthermore, individual treatment by pediatricians was provided in only 23.1% (six out of 26) of respondents. Notably, two respondents reported inpatient treatment, suggesting that a subset of patients experiences significant difficulty recovering at home.

In addition to various mental health issues, behavioral problems such as violence are also observed in GD [10], indicating that such patients may also seek treatment in general psychiatry. Therefore, we asked psychiatrists about their experience of treating GD. Only 52.2% (12 out of 23) of them reported having treated patients with GD, and treatment was primarily provided on an individual basis (30.4%). Additionally, 17.4% (four out of 26) of respondents collaborated with psychologists, reflecting the common presence of clinical psychologists in psychiatric hospitals. Two institutions offered group therapy, and one provided day care services for GD. However, none of the respondents offered support programs for family members. Given that family members may experience significant stress, such programs should be introduced in the near future.

Both pediatricians and psychiatrists reported that the most common clinical symptoms of GD were disturbed sleep-wake rhythm and school refusal/absenteeism, followed by poor academic performance. An early symptom of excessive gaming or internet overuse is reported to be sleep problems [11]. A link between problematic internet use, including GD, and school refusal has also been reported [12]. These results suggest that many parents are particularly concerned about the negative impact of gaming on their children's studies, especially during adolescence. Psychiatric symptoms such as depression and anxiety, as well as behavioral problems like yelling and violence, were also frequently observed among adolescents with GD [13-15]. It is well known that a disrupted sleep-wake rhythm is one of the early symptoms of excessive gaming. Kawabe et al. reported that sleep problems during adolescence can contribute to academic decline and mental health issues [11]. We believe that parents should recognize the importance of adequate sleep and utilize parental controls to regulate gaming duration. For this purpose, collaboration with telecommunications companies will be necessary to educate the public.

The only significant difference between pediatricians and psychiatrists was in their reports of financial issues. Problems related to high in-game spending, such as those associated with loot boxes and microtransactions, have become a growing concern [16,17]. Some adolescent patients with GD resort to stealing money from family members to fund their excessive in-game purchases, leading families to seek medical consultation when they feel unable to manage the issue at home.

When we inquired about psychiatric comorbidities of GD, we found that, consistent with previous studies, NDD such as ASD and ADHD were frequently reported [8]. In particular, ADHD is often associated with GD [18,19]. Pediatricians and CAPs who provide continuous medical support to patients with ADHD from school age to adulthood should be aware of the high risk of developing GD in this population.

Depression and anxiety were the next most frequently reported symptoms. Given that GD is common in adolescents, it is likely that these psychiatric symptoms - both of which are more prevalent during adolescence - are also frequently comorbid [20]. Since gaming and depression are known to coexist [13,21], and depression can be both a risk factor for and a consequence of GD, assessing depressive symptoms is crucial when addressing GD.

A comparison between pediatricians and psychiatrists revealed that only anxiety was reported significantly more often by psychiatrists. In Japan, the school refusal rate among junior high school students is as high as 6.7% [22], and Hikikomori (a severe form of social withdrawal), which often follows school refusal, is a major social concern [23]. Many cases of school refusal and Hikikomori in late adolescence are often diagnosed as anxiety disorders, such as social anxiety disorder, which has been linked to GD [24]. In Japan, pediatricians typically manage patients first diagnosed before the age of 15. Since anxiety disorders are more common in high school students and young adults, this may explain why general psychiatrists observe a higher rate of comorbid anxiety disorders in patients with GD. Pediatricians may be less likely to recognize psychiatric symptoms than psychiatrists. Since GD often manifests in childhood, pediatricians and psychiatrists should have opportunities to learn from one another. Additionally, a system should be established to ensure that cases requiring specialized psychiatric treatment can be referred promptly.

This study has several limitations. First, it was conducted in a specific region, Sapporo, and its findings may not be generalizable to Japan as a whole. We are planning a national survey in the future. Second, the response rate was low, at approximately 30%. The most effective way to increase the survey response rate on GD would be to enhance the participants' interest in GD. Third, the ICD-11 diagnostic guidelines for GD have only been in effect since January 2022, and physicians may still have varying interpretations of the diagnosis. Fourth, the potential influence of confounding factors, such as the respondents' years of clinical experience, has not been thoroughly explored in this study. Fifth, since the response rate was 30.6%, there is a possibility of non-response bias. The characteristics of respondents and non-respondents were not compared, so the generalizability of the findings should be interpreted with caution. Sixth, there are no reliable studies on the prevalence of GD, and the lack of experience treating GD reported by many pediatricians and psychiatrists in this survey may simply suggest that GD is relatively rare. As a result, there may have been inconsistencies among respondents in determining whether to diagnose GD.

## Conclusions

Gaming is a popular pastime among children. With the rise of online gaming and increasingly sophisticated content, playtime has gradually increased, leading to a suspected rise in GD cases. While many CAPs are already addressing game-related issues, only about half of pediatricians and psychiatrists are involved in such treatment. As the number of children and adolescents seeking medical care for gaming-related problems is expected to grow, it is essential to develop guidelines that enable both pediatrics and psychiatry to effectively manage these cases.

## Figures and Tables

**Figure 1:**
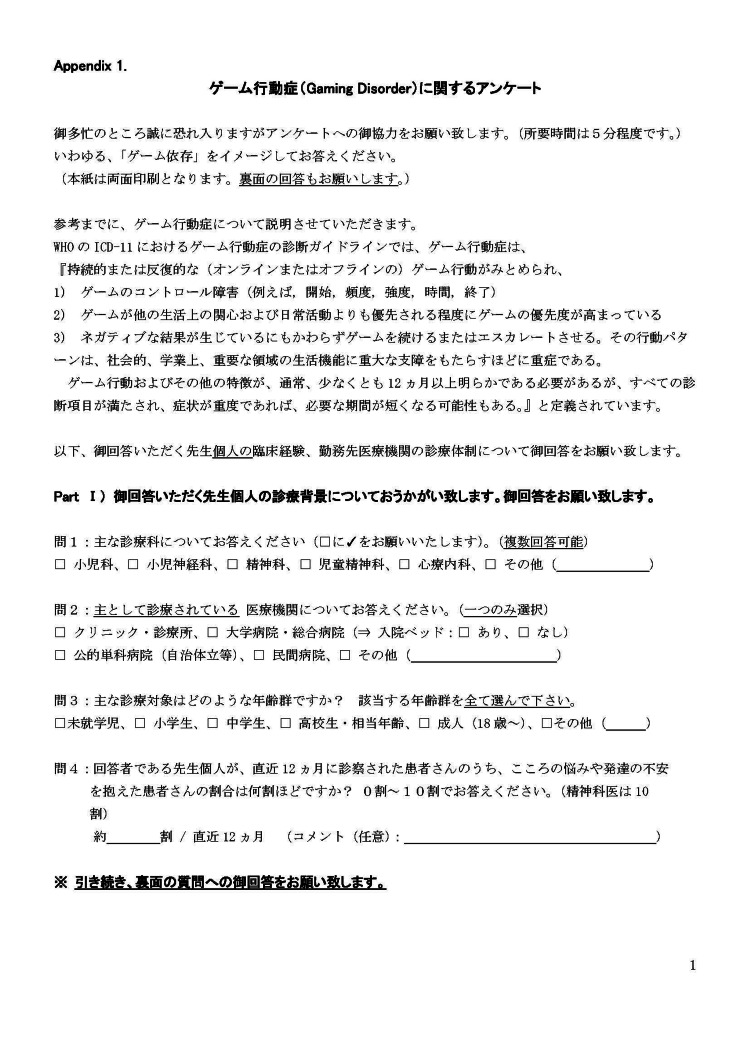
Questionnaire in Japanese (page 1)

**Figure 2:**
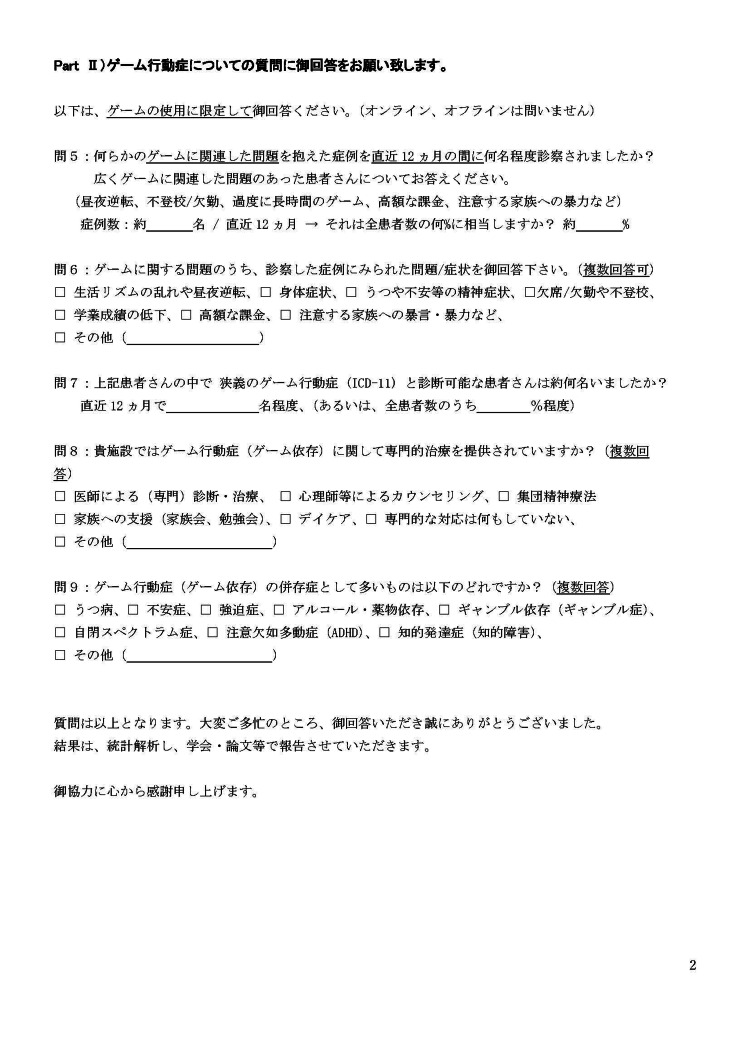
Questionnaire in Japanese (page 2)

**Figure 3:**
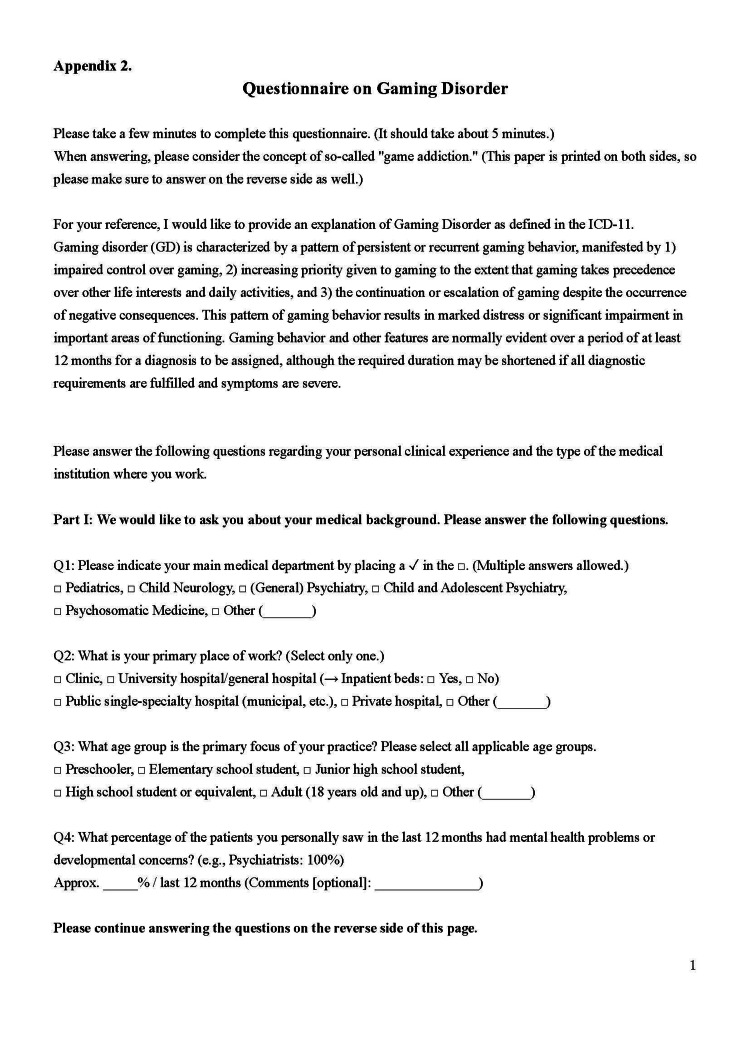
Questionnaire in English (page 1)

**Figure 4:**
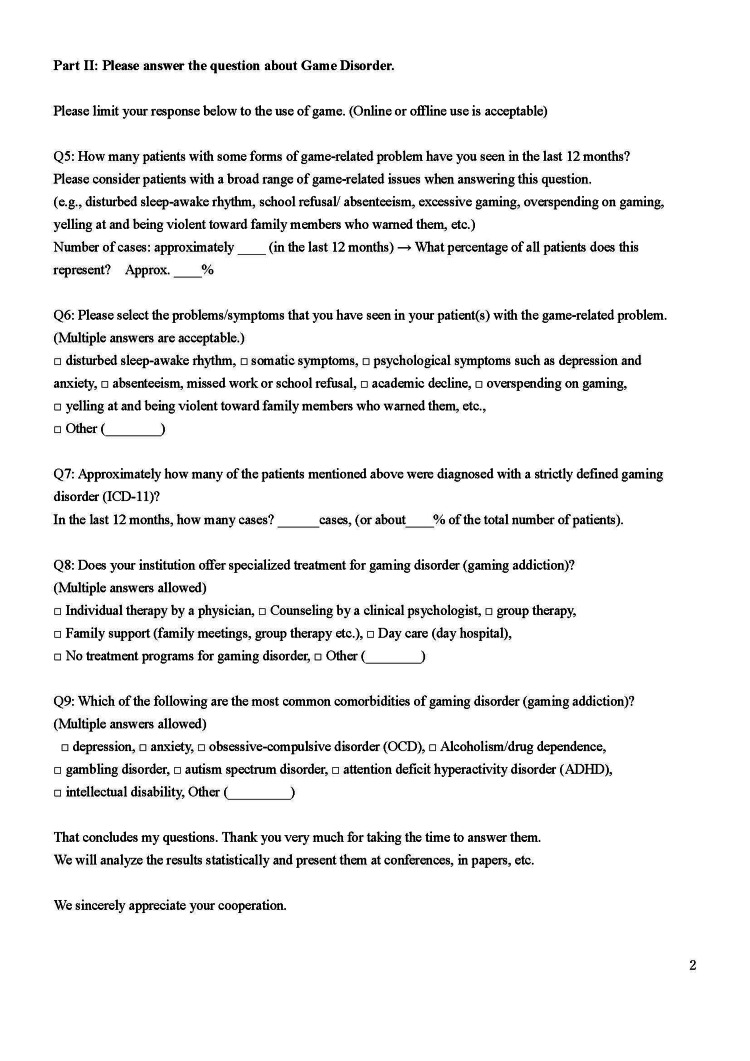
Questionnaire in English (page 2)

## Appendices

Appendix 1: Questionnaire in Japanese

Appendix 2: Questionnaire in English
